# Ser170 of *Bacillus thuringiensis *Cry1Ab δ-endotoxin becomes anchored in a hydrophobic moiety upon insertion of this protein into *Manduca sexta *brush border membranes

**DOI:** 10.1186/1471-2091-10-25

**Published:** 2009-10-19

**Authors:** Oscar Alzate, Craig F Hemann, Cristina Osorio, Russ Hille, Donald H Dean

**Affiliations:** 1 Biochemistry Department, The Ohio State University, Columbus, 43210; OH, USA; 2 Biophysics Program, The Ohio State University, Columbus, 43210; OH, USA; 3 The Davis Heart and Lung Research Institute, The Ohio State University, Columbus, 43210; OH, USA; 4 Department of Biochemistry, University of California, Riverside, 92526; CA, USA; 5 School of Medicine, Cl 78B, # 72A-109, Universidad Pontificia Bolivariana, Medellín, Colombia; 6 Department of Cell and Developmental Biology, School of Medicine, CB 7090, University of North Carolina, Chapel Hill, 27599; NC, USA

## Abstract

**Background:**

Three spin-labeled mutant proteins, mutated at the beginning, middle, and end of α-helix 5 of the *Bacillus thuringiensis *Cry1Ab δ-endotoxin, were used to study the involvement of these specific amino acid residues in ion transport and to determine conformational changes in the vicinity of these residues when the protein was translocated into a biological membrane.

**Results:**

Amino acid residue leucine 157, located in the N-terminal portion of α-helix 5, showed no involvement in ion transport, and the environment that surrounds the residue did not show any change when transferred into the biological membrane. Serine 170, located in the middle of the α-helix, showed no involvement in ion transport, but our findings indicate that in the membrane-bound state this residue faces an environment that makes the spin less mobile, as opposed to the mobility observed in an aqueous environment. Serine 176, located in the C-terminal end of the α-helix 5 is shown to be involved in ion transport activity.

**Conclusion:**

Ion transport data for L157, S170, and S176, along with the mobility of the spin-labels, structural characterization of the resulting proteins, and toxicity assays against a target insect, suggest that the toxin undergoes conformational changes upon protein translocation into the midgut membrane. These conformational changes result in the midregion of the α-helix 5 being exposed to a hydrophobic-like environment. The location of these three residues in the toxin suggests that the entire α-helix becomes inserted in the insect midgut membrane.

## Background

Identification of the amino acid residues involved in the ion transport activity of *Bacillus thuringiensis *(*Bt*) δ-endotoxins remains an interesting challenge. The δ-endotoxins are insecticidal crystal proteins (ICPs) produced by the bacterium during the sporulation stage [1]. The ICPs are composed of several proteins (also known as Cry toxins) with insecticidal activity against insects of the orders Lepidoptera, Diptera and Coleoptera. The ingested crystals are dissolved in the insect midgut to release the protoxin form of the proteins. The action of proteases in the insect midgut cleaves the protoxin at specific locations, releasing the toxic fragment of the protein. In the case of the lepidopteran-specific Cry1A δ-endotoxins, the protoxins have a molecular weight of approximately 140 kDa, while the molecular weight of the toxic fragment is approximately 65 kDa [1].

The toxic action of the protein is initiated by the binding of the active fragment to receptors located on the membrane lining the insect midgut. It has been suggested that after binding to the receptors, the toxin induces ion pores capable of disrupting the membrane potential [2,3]. The molecular events occurring between binding and intoxication remain unclear. Some authors proposed that the binding to these receptors triggers conformational changes that allow the protein to translocate into the membrane, becoming inserted into the lipid moiety [4,5]; such conformational changes, and the membrane bound state of the toxin have not yet been elucidated.

Cry1Ab δ-endotoxin is one of the many insecticidal toxins expressed by *Bacillus thuringiensis*. The three dimensional structure of Cry1Aa (a protein with 89% sequence homology to Cry1Ab), is composed of three structural domains [4]. Domain I is formed by an α-helix bundle formed by seven helices with the α-helix 5 located in the core of the domain. Domains II and III are mainly composed of β-sheets. There is evidence that at least α-helix 7, located in Domain I, is involved in the ion transport activity [6,7]. Domains II and III are involved in receptor recognition in the insect midgut [8,9].

The insertion of the toxin into lipid membranes has been studied using artificial phospholipid vesicles, lipid bilayers [10,11], modified protein toxins in artificial membranes [3], and artificial peptides resembling structural motifs of the protein [12,13]. The protection of δ-endotoxins against proteinase K (Pk) digestion in the presence of brush border membrane vesicles (BBMV) has been used to study the insertion process of the toxin into systems resembling the midgut environment [14-17].

**Figure 1:**
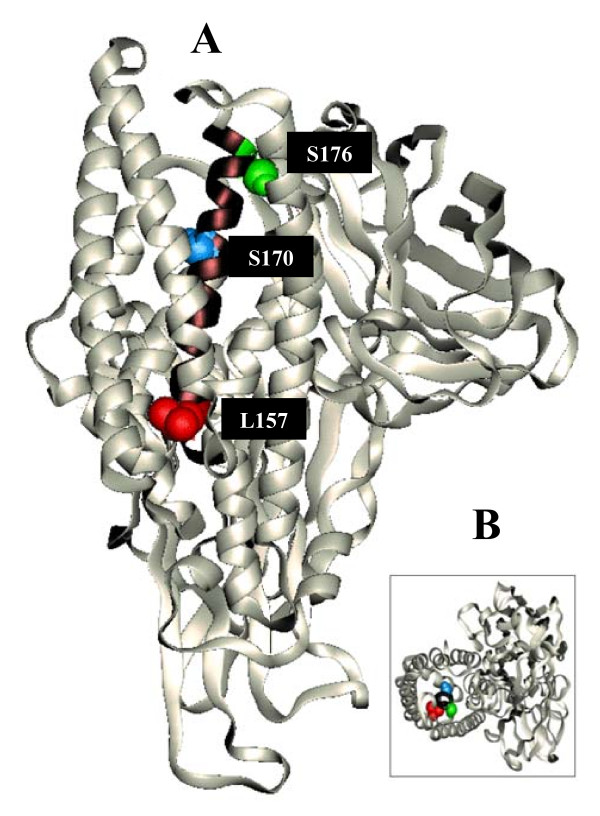
**Three-dimensional representation of *B. thuringiensis *Cry1Ab δ-endotoxin showing the mutations introduced in α-Helix 5 (black in the figure)**. L157 is located at the N-terminal end of the helix (red spheres), S170 in the middle of the helix (blue spheres) and S176 at the C-terminal end (green spheres). The inset shows a view of the toxin from top of the α-helical domain to indicate the central location and the orientation of the amino acid residues mutated for this study.

Site-directed spin-labeling (SDSL) in combination with electron paramagnetic resonance (EPR) spectroscopy is a powerful technique for analyzing the dynamic and structural properties of membrane proteins [18]. To use this technique, specific amino acid residues are mutated into cysteine residues, and then labeled with paramagnetic reagents using the introduced cysteine as the reactive site [19]. For the present study we have used site-directed mutagenesis to introduce single cysteine residues at three positions in the α-helix 5 of *Bt *Cry1Ab (Figure 1). Mutant toxins L157C (N-terminal region), S170C (centrally located) and S176C (C-terminal region) were studied by SDSL, voltage clamping (Vc) and circular dichroism (CD) spectroscopy, among other techniques. Our results indicate that only the C-terminally-located spin-label has an effect in the ion transport activity of the toxin in *Manduca sexta *(*M. sexta*) larvae, without any effect on the structure or the structural stability of the toxin, while the central hydrophobic face of the α-helix becomes inserted in the membrane or another hydrophobic environment that decreases the mobility of the spin-label.

## Results

### Expression of proteins in *E. coli*, purification and bioassays

**Figure 2:**
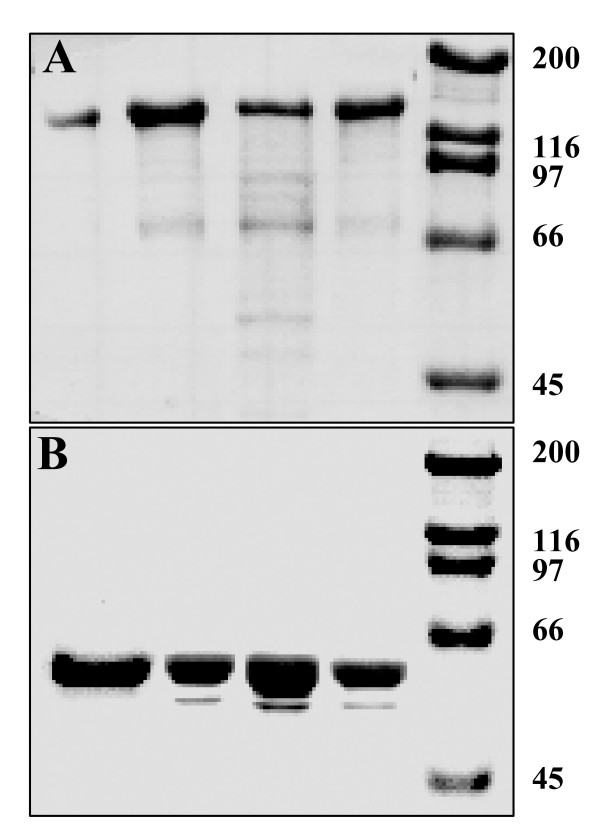
**SDS-PAGE analysis of wild type and mutant toxins**. Panel A, shows the protoxins, and panel B shows the toxins. Lane 1, Cry1Ab wild type; lane 2, L157C; lane 3, S170C; lane 4, S176C and lane 5, molecular weight marker.

Each of the mutations produced protoxins, as determined by SDS (10%)-PAGE (Figure 2, panel A). Tryptic digestion of the protoxins resulted in an active toxic fragment with a size identical to that from wild type Cry1Ab (Figure 2, panel B). Toxicity assays indicate that the toxicity level of S170C (LC_50 _= 40.24 ng/cm^2^) is almost identical to Cry1Ab wild type (LC_50 _= 36.00 ng/cm^2^) with overlapping confidence limits (see Table 1). Mutant toxins L157C and S176C exhibit decreased toxicities by a factor of 2- and 1.5-fold, respectively. The confidence limits for all three toxins overlapped, however, indicating that there were no significant changes in toxicity as determined by this assay. These results, which showed similar toxicities when the confidence limits were considered, did not agree with the results obtained by Vc, as shown in Table 1. The correlation between toxicity and the location of the residue in the α-helix 5 was more interesting when the bioassays were performed with the cysteine residue blocked with the MTS spin-label. In this case, S170R_1 _(LC_50 _= 42.61 ng/cm^2^) and L157R_1 _(LC_50 _= 101.75 ng/cm^2^) showed no difference from the non-labeled mutant protein. The toxicity of the S176R_1 _(LC_50 _= 130.68 ng/cm^2^) mutant decreased by more than two-fold compared to the non-labeled mutant toxin, indicating that the spin-label at C176 affected the toxicity of the protein.

**Table 1:** Toxicity assays and voltage clamping parameters

**Toxin**	**^a^LC_50 _ng/cm^2^**	**^b^Slope, μA/min**	**^c^T_0 _min**.
Cry1Ab	36.00 (19.9-57.8)	-13.42 ± 0.70 -10.93 ± 0.27^d^	4.00 ± 0.14 4.22 ± 0.12^d^

L157C	78.60 (51.3-143.3)	-7.03 ± 0.22	7.58 ± 0.60

L157R_1_	101.75 (95.65-107.85)	-4.27 ± 0.80	8.04 ± 0.20

S170C	40.24 (27.17-56.16)	-11.85 ± 0.60	3.93 ± 0.60

S170R_1_	42.61 (31.76-56.31)	-10.73 ± 0.20	3.08 ± 0.60

S176C	62.15 (34.00-92.90)	-5.56 ± 0.10	6.84 ± 0.15

S176R_1_	130.68 (125.06-136.75)	-3.39 ± 0.12	14.00 ± 0.10

^a^: 50% Lethal doses concentration, numbers in parenthesis represent 95% confidence limits.

^b^: Slope: was obtained by fitting the straight portion of the curve to a linear equation.

^c^: T_0_, defined as the insertion time. This was calculated with equation (1).

^d^: Cry1Ab wild type toxin in the presence of MTS spin-label.

### Voltage clamping

**Figure 3:**
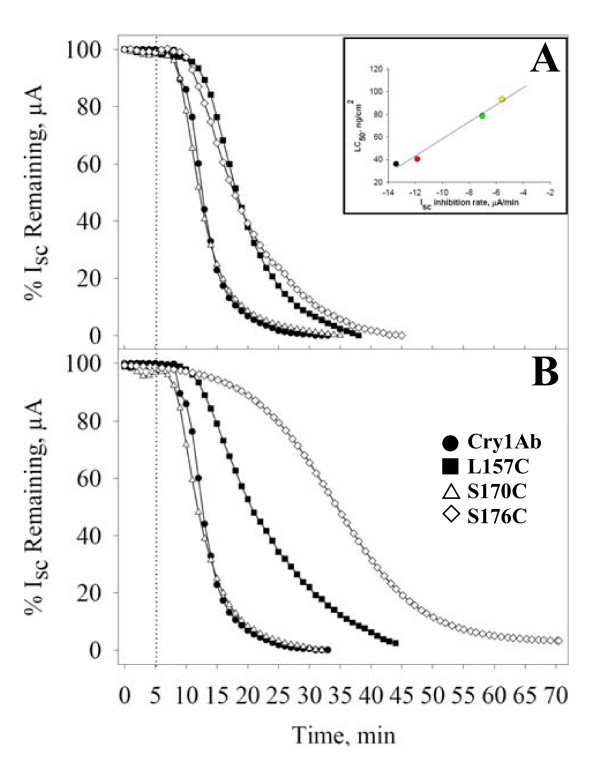
**Voltage Clamping Analysis**. Panel A, wild type Cry1Ab protein and cysteine mutants without MTS-SL. Panel B, wild type protein and cysteine mutants with the cysteine reacted with MTS-SL. The dashed line shows the time when each toxin was added to the chamber. The inset shows the slope calculated from equation (1), plotted against the toxicity determined with the bioassays; indicating that there is a direct correlation between toxicity levels and ion channel activity for these toxins.

Voltage clamping analysis was performed to determine whether the introduced mutations affected the ion transport activity of the toxin, which in turn is directly correlated with toxicity ([6], see inset in Figure 3A). The LC_50 _values for L157C and S170C have similar values to L157R_1 _and S170R_1_, respectively, as shown above. Similar results were seen when the Vc experiments were performed with spin-labeled toxins, indicating that the labeling of these two residues does not affect their toxic activity (Figure 3). When mutant toxin S176C was assayed without the MTS-SL, it showed a decrease in the insertion time (T_0 _= 6.84 ± 0.15 min) compared to the wild-type Cry1Ab (T_0 _= 4.00 ± 0.14 min), and the ion transport activity was slower (as determined by the slope of I_sc _inhibition versus time, see inset in Figure 3A), in accordance with a two-fold decrease in toxicity. When the toxicity produced by this protein was assayed with the spin-label attached to the protein, the insertion time was significantly longer (T_0 _= 14.0 ± 0.15 min) and the ion transport was even slower (slope = -3.39 ± 0.12 μA/min), compared with the wild-type toxin (slope = -13.42 ± 0.70 μA/min) and the non-labeled S176C mutant toxin (slope = -5.56 ± 0.10 μA/min).

The changes seen in the insertion time and in the ion transport activity are likely to occur as a result of capping the cysteine residue with the spin-label since such changes were not found in the other two mutant proteins. The mutant toxin S170C showed the same ion transport activity either with or without capping of the cysteine residue (see Table 1), and these parameters (slope = - 11.85 ± 0.60 μA/min, T_0 _= 3.93 ± 0.60 min) are similar to those of the wild type toxin (slope = -13.42 ± 0.70 μA/min, T_0 _= 4.00 ± 0.14 min). On the other hand, mutant toxin L157C showed a small decrease in ion transport that reflects the decrease observed in LC_50 _with no observable change in the insertion time (see Table 1). The parameters obtained for L157C mutant protein suggest that this toxin has a reduced ability to transport ions (slope = -7.03 ± 0.22 with no MTS-SL and slope = - 4.27 ± 0.80 with MTS-SL, with no change in the insertion time). The results obtained from the voltage clamp experiments are in agreement with the relative toxicities calculated from the toxicity assays (see inset in Figure 3A). These results suggest that the change in slope and insertion time (*i.e*., toxicity) observed for S176C is due to the particular location of the S176 residue, which is therefore critical for ion transport or for partitioning of the toxin in the membrane.

### Circular dichroism spectroscopy and thermal analysis

**Figure 4:**
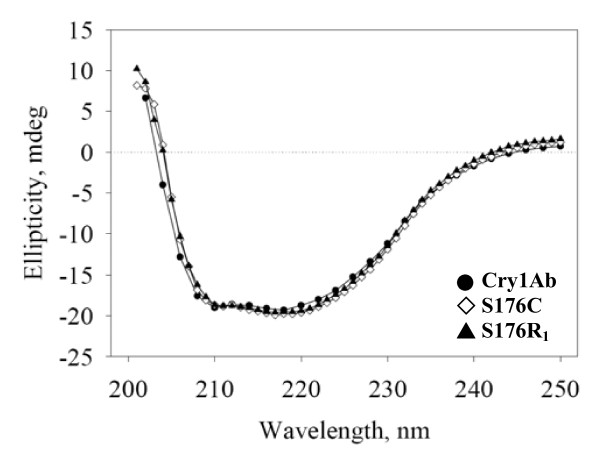
**Circular dichroism analysis of Cry1Ab, S176C and the spin-labeled mutant protein, S176R_1_**.

**Figure 5:**
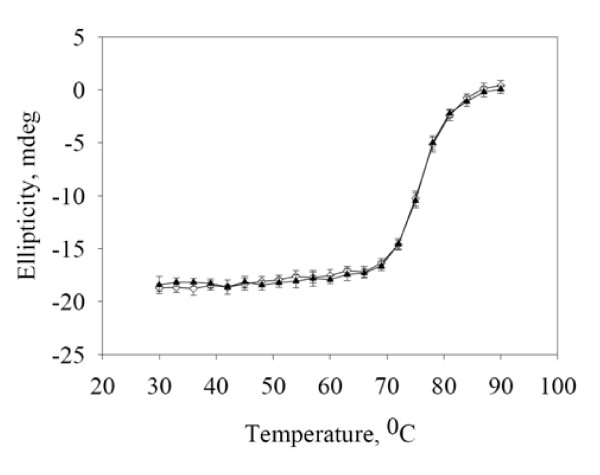
**Thermal denaturation analysis of mutant toxin S176C and the spin-labelled mutant protein, S176R_1_**.

To determine whether structural and stability changes were introduced into the secondary structure of the toxin, either by the mutation or the spin-label, thermal and the CD spectral analyses of S176C were performed (Figure 4). It can be seen that the secondary structures of S176C and S176R_1 _are identical to the secondary structure of the wild-type toxin, with characteristic absorbance peaks at λ = 208 and 220 nm, indicating that neither the mutation, nor the spin-label introduced any observable changes in the secondary structure of the toxins. The thermal stability analysis (shown in Figure 5) shows a typical unfolding curve for Cry toxins, with a very stable native population that starts showing signs of unfolding above 65°C and reaches a fully unfolded population at approximately 90°C, with a T_m _≈ 76°C.

### EPR spectroscopy in solution and in BBMV

**Figure 6:**
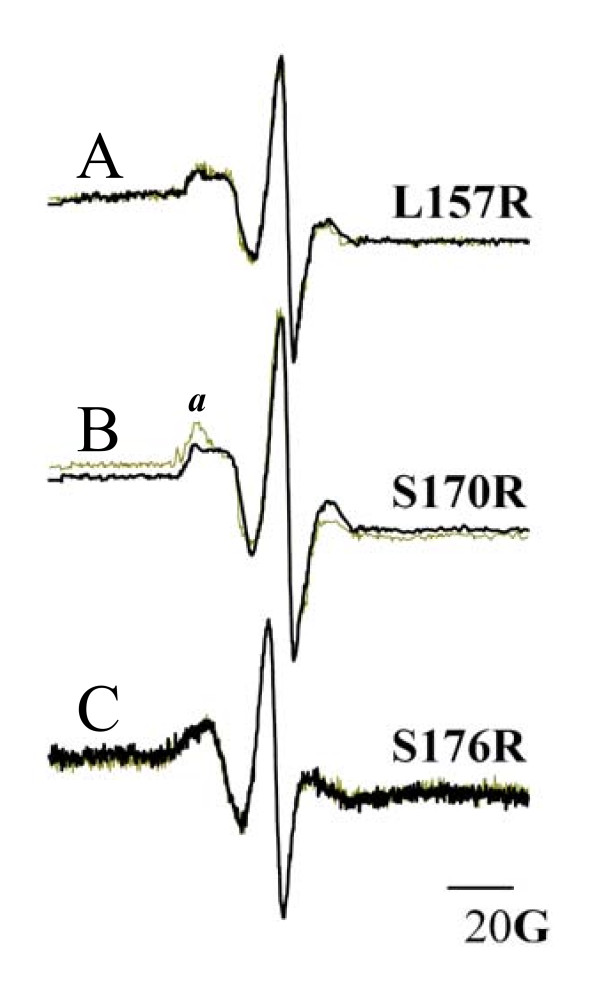
**EPR spectral analysis of the spin-labelled mutant toxins L157C, S170C and S176C in solution and in the membrane**. The dashed line represents the spectra in solution, while the dark line shows the spectra in the membrane. The spectra L157R_1 _and S176R_1 _in the membrane overlap with the corresponding spectra in solution. The spectra for S170R_1 _show small variations in the membrane-bound state, with clear narrowing at position *a*.

The EPR spectrum of a spin-label is sensitive to the environment surrounding the nitroxide [18-22]. For this reason, a spin-labeled protein in solution is expected to display a different EPR signal than one in a hydrophobic environment, either in a biological membrane or a protein core, assuming that the conditions surrounding the nitroxide change from one environment to the other. In the present work we analyzed the EPR spectra of the mutant toxins in solution and in brush border membrane vesicles prepared from *M*. *sexta *midgut tissue. Spectra of each spin-labeled protein in solution were taken at room temperature in a flat cell. Each sample was then transferred to a 1.5 mL vial containing 2.0 mg of BBMV in 250 μL binding buffer, where the mixture was incubated for 45 min at room temperature and transferred back into the cell. The new spectrum was recorded and compared with the data acquired in solution. The EPR spectra for L157R_1_, S170R_1 _and S176R_1 _are shown in Figure 6. The spectra for L157R_1 _and S170R_1 _reflect an environment of low mobility, both in the membrane and in solution. The shapes of these spectra agree with the location of the corresponding amino acids in the protein (Figure 1). The spectrum for S176R_1 _displays a more mobile spin-label, which is also in agreement with the location of the spin-label in the external end of domain I. The spectra of the labeled toxins in the membrane show that *only *the S170R_1 _mutant has changed to a less mobile state in the membrane-bound configuration, although almost no change is observed in the toxicity parameters or the ion transport abilities of the toxin. The insertion of the spin-labeled toxin in the membrane was confirmed by performing bioassay and Vc experiments with spin-labeled toxins. The EPR spectra of the spin-labeled proteins in solution and in the membrane-bound state were analyzed. The results are shown in Figure 6. It is seen that the spectra of L157R_1 _and S176R_1 _in solution are identical to those in the membrane, indicating that there were no changes in the environment of the nitroxide (Figures 6A and 6C). In other words the nitroxide environment was transferred intact from the solution into the membrane. In contrast, the EPR spectrum for S170R_1 _indicates that the nitroxide is *less *mobile when the toxin is inserted into the membrane (Figure 6B).

**Figure 7:**
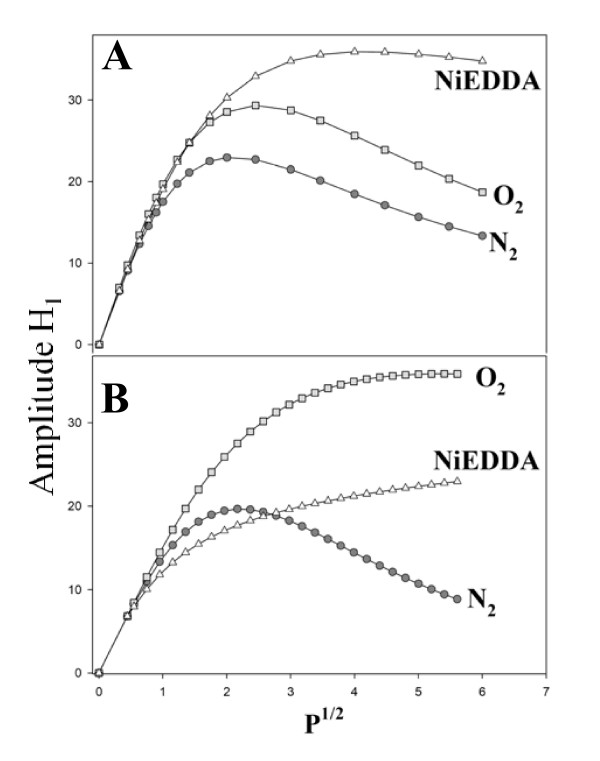
**CW power saturation for S176R1 mutant toxin in A) Solution and B) in *M*. *sexta *BBMV, in the presence of nitrogen, oxygen and NiEDDA, as indicated**.

Continuous wave (CW) power saturation was used to examine whether the S176R_1 _protein was able to penetrate the membrane, as was suggested by the ion transport properties of the mutant. The results, shown in Figure 7, indicate that in the membrane-bound state (Figure 7B) spin exchange between the oxygen and the nitroxide is larger than the exchange with NiEDDA, suggesting that the spin-labeled residue becomes inserted in the membrane. This is in contrast with solution conditions, where exchange with NiEDDA is larger than with oxygen (Figure 7A), suggesting exposure of the nitroxide to the aqueous environment in agreement with the solution structure of the protein. The information presented above suggests that serine 176 is involved in the ion transport capabilities of the toxin, although the exact location of this residue with respect to the resulting pores has not yet been determined. Based on the calibration curve published by Oh et al. [23] our data suggest that the depth of the spin-label from the membrane surface is between 4.0 and 5.0 Å. These results, however, are not conclusive due to the presence of membrane proteins, carbohydrates and a great variety of phospholipids in the BBMV. Thus a calibration curve to determine distances in BBMV should be constructed if the scanning of additional spin-labeled proteins is intended.

## Discussion

Studies on the mode of action of the Bt δ-endotoxins have shown that this is a rather complex mechanism that involves the identification of the target tissue by the activated toxin, followed by association to membrane receptors and internalization into the membrane. The regions of the toxin responsible for membrane internalization and ion transport, and the active conformation of the toxin in the membrane, have not yet been determined. In the present work, we used a combination of site-directed spin-labeling and voltage clamping to address these issues.

Three amino acid residues located in the postulated ion-transport region were individually mutated to cysteine. Each resulting toxin was tested for structural stability and biological activity. Amino acid residue L157 is located at the N-terminal end of the centrally-located α-helix 5, S170 is located at the middle, and S176 at the C-terminal end of the helix (Figure 1). The three mutated proteins produced stable protoxins with the same size as the wild type toxin (Figure 2A); after tryptic digestion, each protoxin released the stable toxin of the same size as the wild type Cry1Ab (Figure 2B). The biological effect of each introduced mutation was first analyzed by bioassays on *M*. *sexta *larva. Results show that S170C has the same toxicity as the wild type toxin while L157C and S176C show a small reduction (about 2-fold decrease). Although the LC_50_values for L157C and S176C clearly show that these two proteins are less toxic than the wild type, the overlapping confidence limits indicate that these bioassays are not accurate enough to determine toxicity differences within such a close range.

To determine more accurately the effects of the mutations on the toxicity, we analyzed the ion transport activity of the toxins by voltage clamping. Our results showed that mutation S170C had no effect at all in ion transport, while mutations L157C and S176C decreased the ion transport activity of the toxins (Figure 3A). Calculated values from the voltage clamping curves are shown in the table. The toxins were compared by analyzing their slopes and insertion times [6,14]. Both the slope and the insertion time for S170C were almost identical to those of the wild type, while these parameters indicated a reduction in ion transport and insertion time for L157C and S176C. The accuracy of the voltage clamp was reflected in the correlation between the LC_50 _values and ion transport, as shown in the insert on Figure 3A, in which a plot of LC_50 _*vs*. slope shows a linear correlation.

To assess the effects of the spin-label on the biological properties of the toxins, each mutant toxin was reacted with MTS-SL, non-attached MTS was removed, and the bioassay and voltage clamping experiments were carried out. The S170R_1 _mutant toxin showed no change either in toxicity or ion transport. The L157R_1 _mutant toxin showed a slight decrease in toxicity, which correlates with extent of loss of ion transport activity. On the other hand, the S176R_1 _mutant showed a large decrease in both the toxicity and the ion transport that are statistically significant. One possible reason why this mutant was less active when the cysteine residue was modified with the spin-label could have been the introduction of large conformational changes due to the bulky side chain. The longer insertion time of the S176R1 mutant could also be due to steric limitations introduced by the modified side chain. Once the protein has been inserted, the ion transport should resemble the activity of S176C provided that the amino acid is not involved in this function. However, the slope of a plot of I_sc _*vs*. time, obtained from voltage clamp experiments, became even less steep suggesting that the amino acid residue at position 176 was involved in ion transport.

CD was used to analyze structural changes in toxin structure. The CD spectra showed that there were no changes in any of the structures, with or without the spin-label. The particular situation of S176R_1_, which showed a considerable decrease in ion transport, indicated that neither the mutation, nor the presence of the spin-label had any effect on the secondary structure (Figure 4). CD thermal melting was used to determine whether the spin-label had introduced any structural instability in the protein. As shown in Figure 5, the thermal melting curve of the S176R_1 _mutant overlaps with the thermal melting curve of the S176C mutant, with a T_m _= 76°C, the same as the wild-type toxin.

By analyzing the conformation of the protein in solution, as shown in Figure 1, it is clear that some conformational changes must accompany the transfer of the protein from solution into the lipid moiety, so the S170 residue becomes located in a more viscous environment that restricts the tumbling seen in solution. One possible explanation, in agreement with two previous publications in which α-helix 5 is proposed to anchor to the membrane moiety [24,25], would be that, after α-helix 1 is cut out of the structure [16], α-helices 2 and 3 open up exposing the hydrophobic face of α-helix 5 to the hydrophobic moiety of the membrane. This would explain why the motion of the modified 170 residue becomes more restricted in the membrane-bound state. It is also possible, however, that location of the spin-label in a hydrophobic environment dominated by hydrophobic amino acids would produce similar observations.

It is clear that the combination of site-directed spin-labeling and voltage clamping allows the determination of functional amino acid residues involved in ion transport activity of the Bt δ-endotoxins, and gives information about the membrane-bound conformation of these important insecticidal toxins. Two very important aspects of this combination of methodologies are, first, that voltage clamping allows for experiments on the midgut tissue containing functional receptors in their native environment and, second, that EPR gives information on the spin-labeled proteins inserted in BBMV. This is in contrast to results obtained from artificial phospholipid vesicles in which the receptors and the structure of the membrane are not necessarily the same as in the biological organism. Along with these advantages, the EPR experiments can also be carried out in artificial phospholipid vesicles. These experiments would allow one to determine the effects of membrane proteins on the EPR spectrum of the spin-labeled toxin.

## Conclusion

**Figure 8:**
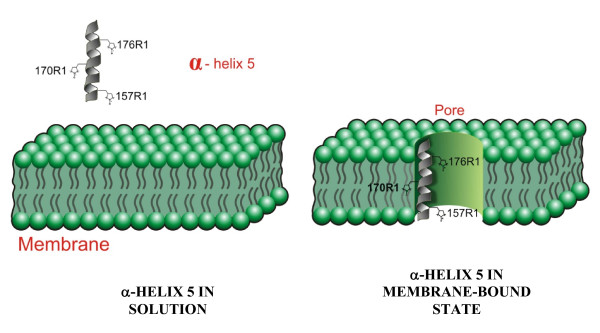
**Model of the membrane-bound α-Helix 5**. The structural transformation suggested by the use of spin-labelled α-helix 5, indicates that this helix participates asymmetrically in the ion channel activity of the toxin. In the membrane-bound state Ser176 is part of the ion pore, while Leu157 is not. In contrast, amino acid Ser170 changes from a mobile to a less-mobile conformation.

It has long been debated whether the different α-helices in domain I of Bt δ-endotoxins undergo conformational changes which allow them to accommodate to the hydrophobic environment of the insect midgut. The data presented here support this hypothesis, and suggest that α-helix 5 spans the membrane with the terminal end-located residues (L157 and S176) facing either the water side of the pore or the aqueous environment outside the membrane, and the mid region residue (S170) facing a hydrophobic environment that restricts the mobility of the spin-label. Since this helix is in the middle of the α-helical bundle, to account for this change in spin-label mobility, this conformational change requires that the whole domain re-accommodates upon protein translocation into the midgut membrane. A possible model for the membrane-bound state of α-helix 5 is shown in Figure 8.

## Methods

### Site-directed mutagenesis, toxin preparation and analysis

Selection of the amino acid residues to be targeted for mutagenesis was based on the Cry1Ab protein structure modeled from Cry1Aa (Pdb code 1CIY), whose crystal structure is available [4]. The coordinates for Cry1Aa were used as template to introduce the changes needed to transform Cry1Aa into Cry1Ab with the protein tools included in the program QUANTA (Molecular Simulations, Inc.). Each mutation was energy minimized to Root Mean Square (R.M.S.) of 0.1 using a Polak-Ribiere algorithm with the force field MM+ (Hypercube, Inc.). Mutant toxins L157C, S170C and S176C were constructed by site-directed mutagenesis as described [26]. The plasmid containing the target mutation was transformed into *E. coli *MV1190 (BioRad) and purified as described [26]. The protoxin form of the proteins was extracted from the inclusion bodies by dissolving the crystals in 50mM carbonate buffer, pH 10.5, at 37°C with constant agitation for 2.0 hours. The presence of the protein was confirmed with SDS (10%)-PAGE gels. Toxins were obtained by digesting the protoxins in 1/20 protoxin/trypsin (w/w) ratio at 37°C for 2.0 hours. The digestion step was repeated under the same conditions. The toxins were purified by size-exclusion chromatography using an Akta Explorer workstation (GE Healthcare, Piscataway, NJ) with a GE Healthcare HiLoad 16/60 Superdex200 column.

### Bioassays

Bioassays and other experimental procedures were performed following guidelines approved by the Institutional Animal Care and Use Committee of the Ohio State University. Toxicity levels were determined with the diet surface contamination assay. For this assay, 24 well sterile dishes (Falcon) were half filled with artificial diet (Bioserv, Frenchtown, NJ). Five dilutions of the toxin were prepared to final concentrations ranging from 1 to 300 ng/cm^2^, and 50 μL of each dilution was poured on the surface of the food. Two (2-3 days old) *M. sexta *larvae were placed in each well and the lethality was scored after five days. The lethal concentration killing 50% of the larvae (LC_50_) was calculated with the program SoftTox (WindowChem Software, Inc.). Each assay was repeated three times.

### Electrophysiology

The inhibition of short circuit current (I_sc_) by toxin in *M. sexta *midguts was measured by voltage clamping (V_c_) following a procedure described by Harvey et al. [27]. Briefly, the anterior portions of early 5th instar *M. sexta *midguts were placed in membrane holders having a surface area of about 0.44 cm^2^. Both sides of the tissue were constantly bathed in oxygenated buffer. The current was measured with a DVC-1000 voltage/current clamp (World Precision Instruments). Twenty μL of toxin (concentration 25 ng/μL) was added on the lumen side of the tissue after the I_sc _current had been stable for approximately 20 minutes. The data recorded in this way were normalized to % of I_sc _remaining and fitted with the equation

(1)

where I_sc _is the short circuit current as function of time, I_sc0 _is the I_sc _value at the time when toxin is injected in the chamber, t_0 _is the time needed for the current to drop 50% from the initial value and A and b are fitting parameters. The time needed for the current to drop 10% from the initial value, as calculated from equation (1), was considered to be proportional to the insertion time, T_0_. Experiments for each toxin were repeated at least three times. The slope corresponding to the I_sc _between 20% and 80% was calculated with the program Sigma-Plot (Jandel Scientific Corporation, Inc.) and was taken to be proportional to the ion-transport activity of the toxin. To test the effect of the introduced cysteine mutations the residues were reacted with MTS as explained below, using EPR spectroscopy.

### Circular dichroism spectroscopy and thermal analysis

Proteins for CD analysis were purified as explained above using 40 mM phosphate buffer, pH 7.4 as mobile phase. Thirty μg of toxin was analyzed in a 1.0 cm path length Hellma quartz cuvette, in the range λ = 200-250 nm at room temperature in an Aviv 62DS spectrometer. The thermal melting analysis was done by increasing the temperature from 30 to 90°C in 3°C increments. The equilibration point was determined by the program IGOR, which controls the CD spectrometer. The CD spectrum for melting analysis was taken at λ = 223 nm.

### EPR Spectroscopy and continuous wave (CW) power saturation

[1-Oxyl-2,2,5,5-tetramethyl-D-pyrroline-3-methyl] methanethiosulfonate (MTS) was from Toronto Research Chemicals (Toronto, Canada). Samples for EPR spectra were purified as described above. After purification the samples were dialyzed against 4,000 volumes of 20 mM Tris-HCl, 1.0 mM EDTA, 150 mM NaCl, pH 7.4 for 4 days with 4 to 5 changes of buffer. After the first day of dialysis, the samples formed a white precipitate that could be easily removed from solution by centrifugation. To spin-label the protein, each sample was mixed with 5.0 molar excess MTS spin-label reagent and allowed to react for 12 hours at room temperature. Excess reagent was removed by dialysis under the same conditions as above, followed by centrifugation at 12,000 RPM for 15 minutes. HPLC analysis, as described, was used to determine that the samples used in all of the different assays were in a monomeric form. Samples were concentrated to 0.1 mM, and a 200 μL aliquot was loaded into a flat cell. Spectra were recorded using a Bruker ESP300 EPR spectrometer at room temperature. The resulting spectra were the average of 20 scans.

For CW power saturation, the NiEDDA was synthesized following a protocol kindly provided by Dr. Jim Feix (Medical College of Wisconsin). The samples were loaded into gas permeable TPX capillaries and the data collected in a loop-gap resonator. The collision parameter Π was measured by power saturation as previously described [20,21], with incident microwave power ranging from 0.1 to 40 mW. Briefly, the peak to peak amplitude (H) of the resonance line as function of the incident microwave power was fitted to equation (2) [20]

(2)

where P is the incident microwave power, P_1/2 _is the power at which H is half of the unsaturated value, ε is a factor related with the homogeneity of the line, and I is a scaling factor [21]. P_1/2 _was determined in the absence of paramagnetic species, in the presence of oxygen and in the presence of 40 mM NiEDDA and the change in P_1/2_, ΔP_1/2_, was determined from these measurements as described by Altenbach et al. [20].

## List of Abbreviations

*Bt*: *Bacillus thuringiensis*; BBMV: brush border membrane vesicles; NiEDDA: Ni (II) ethylenediaminediacetate; EPR: electron paramagnetic resonance; SDSL: site-directed spin-labeling; MTS-SL: [1-oxyl-2,2,5,5-tetramethyl-D-pyrroline-3-methyl]methanethiosulfonate spin-label; Vc: voltage clamping; Pk: proteinase K.

## Authors' contributions

OA conceived the study, designed the experiments, performed the site-directed mutagenesis, purified multiple proteins for the different assays, performed most of the experiments, and prepared the manuscript; CH performed the EPR experiments, helped with EPR data analyses, and helped with manuscript preparation; CO purified proteins for multiple assays, performed the bioassays, and helped with voltage clamping; RH participated in the design of the EPR experiments, and performed EPR data analyses; and DHD directed the experimental design, helped with data analyses, and participated in manuscript preparation. All authors read and approved the final manuscript.

## References

[B1] Schnepf E, Crickmore N, VanRie J, Lereclus D, Baum J, Feitelson J, Zeigler DR, Dean DH (1998). *Bacillus thuringiensis *and its pesticidal crystal proteins. Microbiol Mol Biol Rev.

[B2] Knowles BH, Dow JAT (1993). The crystal δ-endotoxins of *Bacillus thuringiensis*: models for their mechanism of action on the insect gut. BioEssays.

[B3] Schwartz JL, Juteau M, Grochulski P, Cygler M, Préfontaine G, Brousseau R, Masson L (1997). Restriction of intramolecular movements within the CrylAa toxin molecule of *Bacillus thuringiensis *through disulfide bond engineering. FEBS Letts.

[B4] Grochulski P, Masson L, Borisova S, Pusztai-Carey M, Schwartz JL, Brousseau R, Cygler M (1995). *Bacillus thuringiensis *CryIA(a) insecticidal toxin: crystal structure and channel formation. J Mol Biol.

[B5] Li J, Carroll J, Ellar DJ (1991). Crystal structure of insecticidal δ-endotoxin from Bacillus *thuringiensis *at 2.5 Å resolution. Nature.

[B6] Alcantara EP, Alzate O, Lee MK, Curtiss A, Dean DH (2001). Role of α-Helix Seven of *Bacillus thuringiensis *Cry1Ab δ-Endotoxin in Membrane Insertion, Structural Stability, and Ion Channel Activity. Biochemistry.

[B7] Chandra A, Ghosh P, Mandaokar AD, Bera AK, Sharma RP, Das S, Kumar PA (1999). Amino acid substitution in α helix 7 of Cry1Ac δ-endotoxin of leads to enhanced toxicity to *Helicoverpa armigera*. Bacillus thuringiensis.

[B8] Jenkins JL, Lee MK, Valaitis AP, Curtiss A, Dean DH (2000). Bivalent sequential binding model of a *Bacillus thuringiensis *toxin to gypsy moth aminopeptidase N receptor. J Biol Chem.

[B9] Rajamohan F, Alcantara E, Lee MK, Chen XJ, Curtiss A, Dean DH (1995). Single amino acid changes in domain II of Bacillus thuringiensis CryIAb δ-endotoxin affect irreversible binding to *Manduca sexta *midgut membrane vesicles. J Bacteriol.

[B10] Martin FG, Wolfersberger MG (1995). *Bacillus thuringiensis *δ-endotoxin and larval Manduca sexta midgut brush-border membrane vesicles act synergistically to cause very large increases in the conductance of planar lipid bilayers. J Exper Biol.

[B11] Slatin SL, Abrams CK, English L (1990). Delta-endotoxins form cation-selective channels in planar lipid bilayers. Biochem Biophys Res Commun.

[B12] Gazit E, Rocca PL, Sansom MSP, Shai Y (1998). The structure and organization within the membrane of the helices composing the pore-forming domain *of Bacillus thuringien *sis δ-endotoxin are consistent with an "umbrella like" structure of the pore. Proc Natl Acad Sci USA.

[B13] Gazit E, Shai Y (1995). The assembly and organization of the α5 and α7 helices from the pore-forming domain of *Bacillus thuringiensis *δ-endotoxin. J Biol Chem.

[B14] Arnold S, Curtiss A, Dean DH, Alzate O (2001). The role of a proline-induced broken-helix motif in α-helix 2 of *Bacillus thuringiensis *δ-endotoxins. FEBS Letters.

[B15] Aronson A (2000). Incorporation of protease K into larval insect membrane vesicles does not result in disruption of integrity or function of the pore-forming *Bacillus thuringiensis *δ-endotoxin. Appl Environ Microbiol.

[B16] Aronson AI, Geng C, Wu L (1999). Aggregation of *Bacillus thuringiensis *Cry1A toxins upon binding to target insect larval midgut vesicles. Appl Environ Microbiol.

[B17] Himeno M, Ihara H, Clark JM (1995). Mode of action of δ-endotoxin from *Bacillus thuringiensis *var. *aizawai*. Molecular Action of Insecticides on Ion Channels.

[B18] Hubbell WL, Cafiso DS, Altenbach C (2000). Identifying conformational changes with site-directed spin labeling. Nat Struct Biol.

[B19] Hubbell WL, Altenbach C, White S (1994). Site-Directed Spin Labeling of Membrane Proteins. Membrane Protein Structure: Experimental Approaches.

[B20] Altenbach C, Flitsch SL, Khorana HG, Hubbell WL (1989). Structural studies on transmembrane proteins. 2. Spin labeling of bacteriorhodopsin mutants at unique cysteines. Biochemistry.

[B21] Altenbach C, Greenhalgh DA, Khorana HG, Hubbell WL (1994). A collision gradient method to determine the immersion depth of nitroxides in lipid bilayers: application to spin-labeled mutants of bacteriorhodopsin. Proc Natl Acad Sci USA.

[B22] Berliner LJ, Reubens J (1989). Biological Magnetic Resonance.

[B23] Oh KJ, Zhan H, Cui C, Altenbach C, Hubbell WL, Collier RJ (1999). Conformation of the diphtheria toxin T domain in membranes: a site-directed spin labeling study of the TH8 helix and TL5 loop. Biochemistry.

[B24] Masson L, Tabashnik BE, Liu YB, Brousseau R, Schwartz JL (1999). Helix 4 of the *Bacillus thuringiensis *Cry1Aa toxin lines the lumen of the ion channel. J Biol Chem.

[B25] Loseva OI, Tiktopulo EI, Vasiliev VD, Nikulin AD, Dobritsa AP, Potekhin SA (2001). Structure of Cry3A δ-endotoxin within phospholipid membranes. Biochemistry.

[B26] Alzate O, You T, Claybon M, Osorio C, Curtiss A, Dean DH (2006). Effects of disulfide bridges in domain I of Bacillus thuringiensis Cry1Aa δ-endotoxin on ion-channel formation in biological membranes. Biochemistry.

[B27] Harvey WR, Crawford DN, Spaeth DD (1990). Isolation, voltage clamping, and flux measurements in lepidopteran midgut. Methods in Enzymology.

